# Serum levels of IL-12, IL-18, and IL-21 are indicators of viral load in patients chronically infected with HBV

**DOI:** 10.1590/1414-431X2022e12320

**Published:** 2022-11-11

**Authors:** Fangye Zhou, Haoran Xiong, Shenghang Zhen, Aimin Chen, Min Huang, Yupeng Luo

**Affiliations:** 1 Chengdu Fifth People's Hospital, Chengdu University of Traditional Chinese Medicine, Chengdu, Sichuan, China; 2 Department of Clinical Laboratory, Fujian Medical University Teaching Hospital, The First Hospital of Putian, Putian, Fujian, China

**Keywords:** Hepatitis B virus, Interleukins, Chronic hepatitis B virus patients, HBV-DNA loads

## Abstract

This study explored the correlation between interleukins (IL)-12, IL-18, and IL-21 and the viral load in patients with chronic hepatitis B virus (HBV). A total of 142 patients were consecutively enrolled. All were hepatitis B surface antigen (HBsAg)-positive for >6 months and did not receive drug therapy. An ELISA kit was used to test the IL-12, IL-18, IL-21, and acetylcholinesterase (AchE) levels in serum samples from chronic HBV patients and healthy control groups. The amounts of IL-12 and IL-18 were highest in the 5-6log10 (high viral load) group, while IL-21 was highest in the 3-4log10 (low viral load) group. Also, the IL-21 amount was decreased in the HBsAg+/HBeAg/HBcAb+ group, and IL-12, IL-18, and IL-21 were decreased in the normal alanine aminotransferase (ALT) group compared to the abnormal ALT group. These data suggested that IL-12, IL-18, and IL-21 serum levels were positively correlated with disease progression and could reflect disease severity for different HBV-DNA loads. Detection of IL-12, IL-18, and IL-21 levels was found to be helpful for evaluating the degree of liver cell damage and predicting the progression of hepatitis.

## Introduction

Chronic hepatitis B virus (HBV) infection is a serious global health problem. In 2018, an estimated 292 million people were living with chronic hepatitis B. Moreover, dual HBV/hepatitis C virus chronic co-infection is fairly frequent, especially in endemic areas. In addition, some chronic HBV patients also develop liver fibrosis, cirrhosis, and hepatocellular carcinoma (1,2).

HBV is a circular and partially double-stranded DNA with eight genotypes (3). The main transmission routes are blood and mother-to-child transmission (4). High levels of aspartate aminotransferase (AST) and alanine aminotransferase (ALT) within a range of 1000-2000 IU/mL is the hallmark of HBV disease; yet, some chronic patients have normal ALT levels (5,6).

The diagnosis of HBV infection requires the evaluation of the patient's blood for hepatitis B surface antibody, hepatitis B surface antigen, and hepatitis B core antibody. Different clinical stages (acute *vs* chronic infection) seem to be associated with various immune states (7-10). For example, many interleukins (IL), such as IL-21, IL-12, and IL-18, have an important role in regulating HBV infection (11). IL-21 may promote and regulate B cell differentiation (12-15). IL-12 stimulates the production of interferon γ and tumor necrosis factor α from T cells and NK cells (16). IL-18 is a type of inflammasome signaling protein (17). Yet, previous studies have shown different kinds of HBV-DNA loads and HBV antigens and antibodies in chronic infection (18).

HBV DNA quantification, also known as viral load, is the most direct and reliable indicator of viral replication activity. However, several studies have shown that hepatitis viral load alone is not suitable for direct assessment of disease severity in patients. Thus, in this study, we explored the correlation between IL-12, IL-18, IL-21, and viral load in chronic HBV patients. We hypothesized that this method may improve the diagnosis of liver cell damage and predict the development of hepatitis disease.

## Material and Methods

### Participants

A total of 142 patients with chronic HBV admitted to the Chengdu Fifth People's Hospital between January 2018 and March 2019 were enrolled in this study. The patients were recruited consecutively using convenience sampling. The purpose and methods of the study were explained to all of the participants. All patients were HBsAg-positive for >6 months and did not receive drug therapy. We excluded patients with hepatitis C virus or hepatitis D virus co-infection and cirrhosis at baseline. In addition, healthy controls with normal liver function and without any virus infection confirmed by the physical examination center were enrolled.

The study was performed according to the Declaration of Helsinki was approved by the Chengdu Fifth People's Hospital Ethics Committee. Additionally, written consent was obtained from all of the patients.

### Enzyme-linked immunosorbent assays (ELISA)

HBsAg, HBsAb, HBeAg, HBeAb, and HBcAb were assessed using ELISA kits (Cusabio Biotech, Life Sciences Advanced Technologies Inc., USA, catalog numbers: CSB-E04598h, CSB-E07450h, CSB-E11707h, CSB-E09670h) to test the IL-12, IL-18, IL-21, and AchE levels in serum samples from chronic hepatitis B patients and healthy controls.

### HBV-DNA level and genotyping

HBV-DNA level was quantified using the DaAn GENE (China) RealTime HBV assay, in 0.2 mL serum with a lower detection limit of 200 IU/mL. HBV genotype was determined using the DaAn GENE RealTime PCR-based assay.

### Statistical analysis

Data were evaluated using GraphPad Prism 8.0 software (USA). The data are reported as means±SD, and the *t*-test was used to compare the two groups. One-way ANOVA was used for the comparison of four different groups. A P-value of <0.05 was considered to be statistically significant.

## Results

### Patient demographics

The clinical characteristics of chronic hepatitis B patients are summarized in Table 1. One hundred and forty-two patients were divided into three groups, according to HBV-DNA loads. Patients in the 7-8log10 group were younger than the other two chronic hepatitis B groups (P=0.0002 and P=0.0054) and were more likely to present with high HBV-DNA loads. However, there was no difference in white blood cells (WBC), hemoglobin (HGB), and platelet (PLT) levels between the three groups.

**Table 1:** Clinical characteristics of chronic hepatitis B patients.

Parameters	HBV-DNA loads (IU/mL)				P value
	3-4 log10 (n=48)	5-6 log10 (n=47)	7-8 log10 (n=47)	Healthy controls (n=20)	
Age, years	46.33±13.13	44.27±14.43	36.46±12.01	42.53±13.58	0.0029
Gender (males/females)	28/20	26/21	28/19	20/12	-
Genotype, n (%)					
B	6 (12)	5 (11)	7 (15)	0	-
C	36 (76)	39 (84)	38 (81)	0	-
B+C	6 (12)	3 (6)	2 (4)	0	-
WBC (10^9^/L)	5.97±1.62	6.32±1.40	6.22±1.48	6.19±1.59	0.7169
HGB (g/L)	129.83±17.86	128.14±14.84	133.42±14.17	131.40±20.77	0.4604

The data are reported as means±SD or number and percent (ANOVA). WBC: White blood cells; HGB: hemoglobin.

### Comparison of IL-12, -18, -21, and AchE in serum HBV DNA groups

ELISA was used to investigate alterations of IL-12, IL-18, IL-21, and AchE levels in chronic HBV patients with different serum HBV-DNA loads. IL-18 and IL-21 were increased, while IL-12 amount was decreased in the 7-8log10 group compared to the 5-6log10 group (59.66±34.28 *vs* 90.27±59.96 pg/mL, P<0.05). Multiple comparison suggested differences between 3-4log10, 5-6log10, and 7-8log10 groups (266.90±203.53 *vs* 364.83±233.87 pg/mL, P<0.05; 364.83±233.87 *vs* 240.96±134.06 pg/mL, P<0.05).

**Figure 1:**
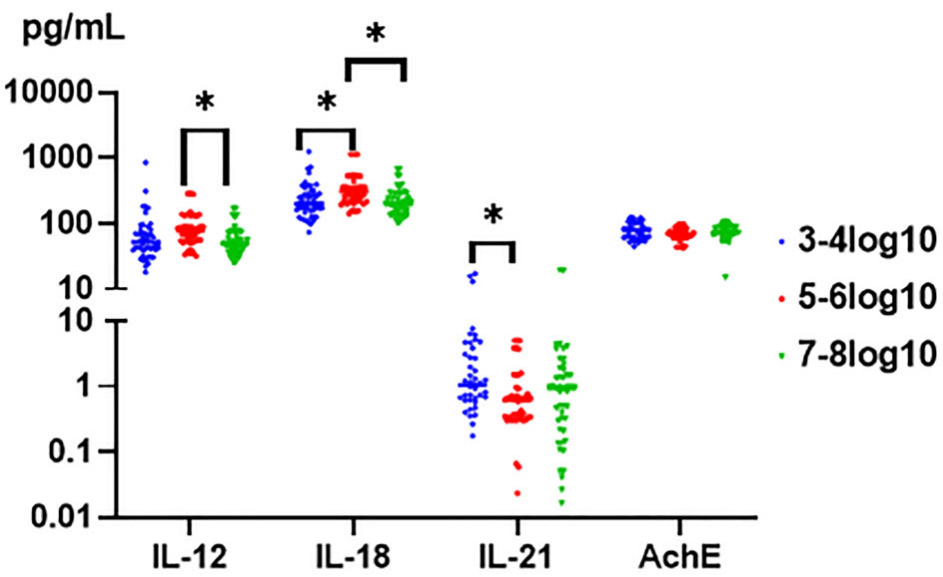
Changes in serological interleukin (IL)-12, IL-18, IL-21, and acetylcholinesterase (AchE) in different serum hepatitis B virus (HBV)-DNA groups. The horizontal line indicates the mean. *P<0.05 (ANOVA).

IL-21 was decreased in the 5-6log10 group compared to the 3-4log10 group (1.03±1.35 *vs* 3.21±5.80 pg/mL, P<0.05). The levels of IL-12, IL-18, and IL-21 were correlated with HBV-DNA loads; the levels of IL-12 and IL-18 were highest in the 5-6log10 group, and the level of IL-21 was highest in the 3-4log10 group. No correlation was found between AchE and HBV-DNA loads (Figure 1).

### Comparison of IL-12, -18, -21, and AchE in serum HBV antigen groups

**Figure 2:**
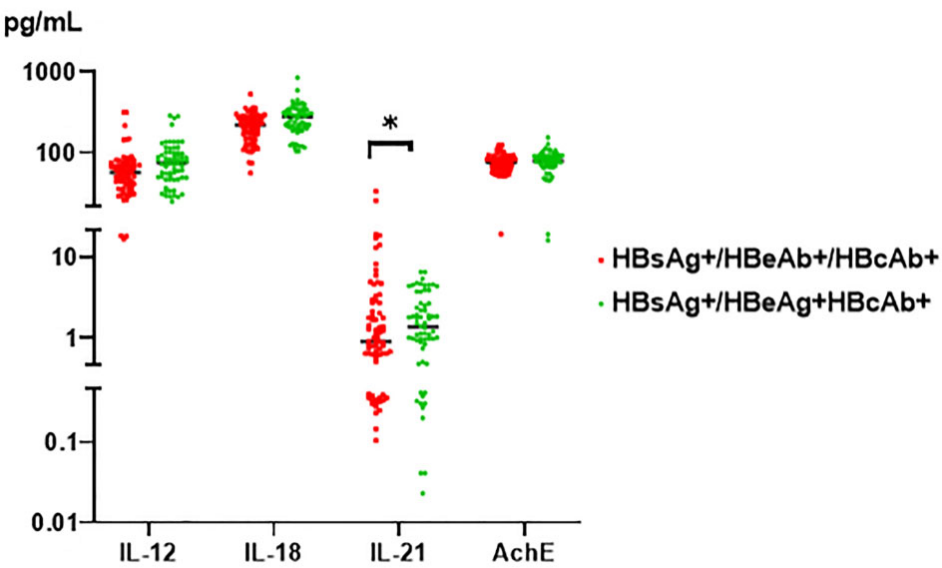
Changes in serological interleukin (IL)-12, IL-18, IL-21, and acetylcholinesterase (AchE) in different serum hepatitis B virus (HBV) antigen groups. The black horizontal line indicates the mean. *P<0.05 (ANOVA).

To further evaluate the regulatory roles of IL-12, IL-18, IL-21, and AchE in chronic HBV patients, serum HBV antigens were determined. According to the positivity of HBsAg, HBsAb, HBeAg, HBeAb, and HBcAb, patients were divided into two groups: HBsAg+/HBeAb+/HBcAb+ group and HBsAg+/HBeAg/HBcAb+ group. The levels of IL-12, IL-18, IL-21, and AchE in the serum were increased, which indicated that they had a major impact on chronic HBV patients (Table 2). In contrast, IL-21 amount was decreased in the HBsAg+/HBeAg/HBcAb+ group compared to the HBsAg+/HBeAb+/HBcAb+ group (1.99±1.74 *vs* 4.27±7.70 pg/mL, P<0.05, Figure 2).

**Table 2:** Comparison of interleukin (IL)-12, IL-18, IL-21, and acetylcholinesterase (AchE) in different serum hepatitis B virus (HBV) antigen groups and healthy controls.

	HBsAg+/HBeAb+/HBcAb+ group (n=76)	HBsAg+/HBeAg+HBcAb+ group (n=66)	Healthy controls(n=20)	P value
HBV-DNA loads (IU/mL)	5.23E+6±1.64E+7	3.37E+7±7.82E+7	-	0.0039
IL-12 (pg/mL)	65.32±51.60	84.28±55.73	29.85±6.48	0.0010
IL-18 (pg/mL)	251.23±196.96	325.40±203.33	78.75±4.89	<0.0001
IL-21 (pg/mL)	4.27±7.70	1.99±1.74	0.69±0.42	0.0085
AchE (mU/mL)	74.83±18.07	78.33±22.25	8.13±2.26	<0.0001

The data are reported as means±SD (ANOVA).

### Comparison of IL-12, -18, -21, and AchE in serum ALT groups

**Figure 3:**
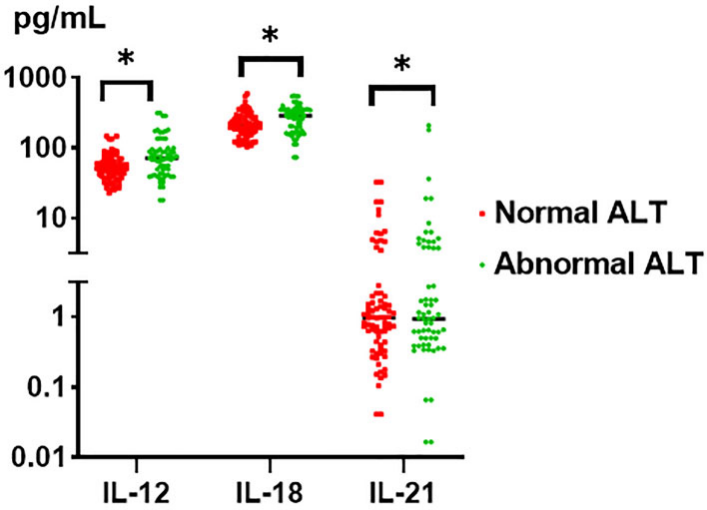
Serological interleukin (IL)-12, IL-18, IL-21, and acetylcholinesterase (AchE) levels in different serum alanine aminotransferase (ALT) antigen groups. The black horizontal line indicates the mean. *P<0.05 (ANOVA).

Normal ALT was defined as <40 U/L. To identify the role of IL-12, IL-18, IL-21, and AchE in chronic hepatitis HBV patients, we compared the levels in different serum ALT groups. The levels of IL-12, IL-18, IL-21, and AchE were significantly increased in different serum ALT groups compared to the healthy group (all P<0.05, Table 3). In contrast, IL-12, IL-18, and IL-21 decreased in the normal ALT group compared to the abnormal ALT group (56.75±26.71 *vs* 90.44±67.85 pg/mL, P<0.05; 228.55±106.36 *vs* 361.63±271.22 pg/mL, P<0.05; 2.84±5.91 *vs* 8.59±3.39 pg/mL, P<0.05, respectively; Figure 3).

**Table 3:** Comparison of interleukin (IL)-12, IL-18, IL-21, and acetylcholinesterase (AchE) in different serum alanine aminotransferase (ALT) groups of different serum hepatitis B virus (HBV) antigen groups and healthy controls.

	Normal ALT (n=76)	Abnormal ALT (n=66)	Healthy controls (n=208)	P value
HBV-DNA loads (IU/mL)	1.62E+7±2.94E+7	2.50E+7±8.32E+7	-	0.3899
IL-12 (pg/mL)	56.75±26.71	90.44±67.85	29.85±6.48	<0.0001
IL-18 (pg/mL)	228.55±106.36	361.63±271.22	78.75±4.89	<0.0001
IL-21 (pg/mL)	2.84±5.91	8.59±3.39	0.69±0.42	<0.0001
AchE (mU/mL)	75.38±19.44	78.03±20.23	8.13±2.26	<0.0001

The data are reported as means±SD (ANOVA).

## Discussion

Assessment of the changes in cytokine expression patterns during various clinical stages of chronic HBV infection may facilitate the understanding of pathogenesis (19,20). According to the level of HBV-DNA loads, hepatitis B virus antigens, hepatitis B virus antibodies, and ALT, we evaluated the serum level of IL-12, IL-18, and IL-21 in chronic hepatitis B virus patients.

It has been reported that the spontaneous HBsAg clearance rate in patients with chronic hepatitis B virus is very low (21,22). However, there are few published data on the relationship between IL levels and viral loads in patients with chronic hepatitis B virus. In this study, the levels of IL-21 in different chronic hepatitis B virus patient groups were significantly different. Therefore, given the serum level of chronic patients, IL levels should be measured in HBV-infected patients with different ALT levels and viral loads.

In conclusion, this study suggested that for hepatitis B virus infection, IL-12 and IL-18 rather than IL-21 were more suitable for assessing disease severity of high viral load (5-6log10) patients and IL-21 was more suitable for patients with low viral load (3-4log10). In addition, the IL-21 levels of HbeAg+ and HbeAb+ patients were higher in the former, so it is meaningful to evaluate the IL-21 level when patients are HbeAg+, while it is meaningless to detect the levels of IL-12 and IL-18 in patients that are HbeAg+ or HbeAb+. Hepatitis viral load alone is not suitable for the assessment of disease severity. The combination of IL-12, IL-18, and IL-21 levels was more helpful to evaluate the degree of liver cell damage and predict the progression of hepatitis.
